# Clinical Application of Oral Meglumine Diatrizoate Esophagogram in Screening for Esophageal Fistula During Radiotherapy or Chemoradiotherapy for Esophageal Cancer

**DOI:** 10.3389/fonc.2020.562147

**Published:** 2020-10-02

**Authors:** Rong Wu, Lidan Geng, Zhenhua Zhao, Dongbiao Liao, Bin He, He Hu, Yanqun Lin, Musheng Li, Miao Xiang, Yu Zhang, Gang Feng, Bangxian Tan, Xiaobo Du

**Affiliations:** ^1^Department of Oncology, Mianyang Central Hospital, Mianyang, China; ^2^Department of Oncology, Affiliated Hospital of North Sichuan Medical College, Nanchong, China; ^3^Department of Oncology, Yan Ting County Cancer Hospital, Yanting County, China; ^4^Department of Radiology, Mianyang Cancer Hospital, Mianyang, China

**Keywords:** esophagogram, esophageal fistula, esophageal cancer, radiotherapy, chemoradiotherapy, meglumine diatrizoate

## Abstract

**Background:** This study aimed to investigate the specificity and sensitivity of oral meglumine diatrizoate esophagogram in screening for esophageal fistula during radiotherapy or chemoradiotherapy for esophageal cancer and determine if early detection and intervention could improve the prognosis of esophageal fistulas.

**Methods:** Esophageal cancer patients undergoing radiotherapy or chemoradiotherapy were included. Weekly oral meglumine diatrizoate esophagograms were performed to screen for esophageal fistulas during radiotherapy. When an esophageal fistula was detected, fibroesophagoscopy and computed tomography (CT) were used for confirmation; once confirmed, radiotherapy was discontinued, and the patient received intervention. The esophagogram results were reviewed weekly to assess the recovery of the esophageal fistula. If the fistula was healed, the patient resumed and completed radiotherapy.

**Results:** A total of 206 patients with cancer of the esophagus undergoing chemotherapy/radiotherapy were included. During radiotherapy, 10 cases of esophageal fistula were detected or suspected based on the oral meglumine diatrizoate esophagography findings, and eight of those cases were confirmed by CT and esophagoscopy. All patients with esophageal fistula received intervention; among them, 62.5% (5/8) recovered after 1 to 2 weeks of treatment and continued radiotherapy to completion. The sensitivity and specificity of oral meglumine diatrizoate esophagography in screening for esophageal fistulas during radiotherapy or chemoradiotherapy were 100 and 98.9%, respectively. The median survival period of patients with esophageal fistulas was 6.4 months.

**Conclusion:** Oral meglumine diatrizoate esophagography has high sensitivity and specificity in screening for esophageal fistulas during radiotherapy or chemoradiotherapy with minimal side effects. Early diagnosis and timely intervention can significantly improve the prognosis and prolong the survival period of patients.

**Trial Registration:** Chictr.org.cn, Identifier: ChiCTR-DDD-17012617. Registered on September 7, 2017. The first participant was enrolled on September 25, 2017. http://www.chictr.org.cn/showproj.aspx?proj=21526.

## Introduction

Esophageal cancer is one of the most common cancers of the digestive system and is associated with a high degree of malignancy and poor prognosis (1). In China, the morbidity and mortality rates of esophageal cancer are ~30 times that of the United States, accounting for the third most common type of malignancy (2). Surgery and chemoradiotherapy are still the main treatment methods for esophageal cancer (3, 4).

Esophageal fistula is one of the most serious complications of radiotherapy treatment for esophageal cancer. More than 5% of patients present with esophageal fistulas, such as esophageal tracheal fistula and esophageal mediastinal fistula. Once a fistula is present, the mortality rate increases significantly, with the median total survival time being only 2 to 3 months (5–9). Fibroesophagoscopy can be used to detect esophageal fistulas; however, it is an invasive examination that can lead to esophageal mucosal damage during radiotherapy. It is also expensive, making it too cost-prohibitive to be suitable. Enhanced spiral computed tomography (CT) can be used to not only observe the integrity of the esophageal wall, but also to clearly observe the surrounding tissues and organs. Additionally, enhanced spiral CT provides for the early detection of small amounts of gas accumulation around the esophagus (10, 11); however, it is too expensive and thus unsuitable for routine screening.

The esophagogram is a non-invasive and inexpensive examination that details the structure and function of the esophageal mucosa and can determine the scope of esophageal lesions. To date, barium esophagogram has been widely used for the screening and diagnosis of esophageal cancer, but it is unsuitable for routine screening of esophageal fistulas. Barium has been shown to cause local mechanical stimulation, inflammation, and persistent or intermittent cough symptoms, all of which affect patients' quality of life. As an alternative to barium, an esophagogram with a water-soluble contrast agent has been recommended for patients with suspected esophageal fistulas because it can be rapidly absorbed from the mediastinum (12).

Meglumine diatrizoate is a water-soluble contrast agent with short half-life that is quickly metabolized; inexpensive, compared to other agents; and rarely adversely reactive in the body cavity. Accordingly, meglumine diatrizoate esophagogram seems to be the most suitable screening method for esophageal fistula in patients undergoing radiotherapy; however, its sensitivity and specificity in diagnosing esophageal fistulas during radiotherapy have not been reported.

The purpose of this study was to investigate the specificity and sensitivity of oral meglumine diatrizoate esophagogram in screening for esophageal fistulas in patients undergoing radiotherapy or chemoradiotherapy for esophageal cancer and to determine if early detection and intervention can improve the prognosis of esophageal fistulas.

## Materials and Methods

The recruitment methods and design of the study, inclusion and exclusion criteria, data collection and management, and sample size calculations have been previously described (13). Patients were selected according to the inclusion and exclusion criteria. Weekly oral meglumine diatrizoate esophagogram was performed to screen for esophageal fistulas. When an esophageal fistula was detected, fibroesophagoscopy and CT were used to confirm it, radiotherapy was subsequently discontinued, and the patient received high intravenous nutrition and anti-inflammatory therapies. The esophagogram was reviewed weekly to assess the patient's recovery from the esophageal fistula. If the oral meglumine diatrizoate esophagogram showed that the fistula was healed, then fibroesophagoscopy was performed to confirm it, and the patient resumed the radiotherapy treatment.

### Radiotherapy

All enrolled patients received intensity-modulated radiotherapy. The gross tumor volume (GTV) was delineated using information from clinical examination and diagnostic imaging and encompassed all overtly macroscopic diseases. Lymph nodes were added to the GTV in case of central necrosis, loss of fatty hilum, or pathologic enlargement (short-axial diameter ≥10 mm or esophageal sulcus lymph nodes ≥5 mm). The clinical target volume (CTV) included sites of tumor invasion and lymphatic drainage where metastasis might occur. The planning target volume (P-GTV and P-CTV) was created by three-dimensional expansion of the GTV and CTV by a margin of 5 mm. The radiation dose was P-GTV 60.2–66 Gy/28–33 fractions and P-CTV 50.4–59.4 Gy/28–33 fractions. Radiotherapy was administered five times a week for a total of 6–7 weeks.

### Chemotherapy

The concurrent chemoradiotherapy scheme includes fluoropyrimidine and platinum; taxus and platinum; tegafur, gimeracil, and oteracil potassium (S1); platinum; and other agents.

### Statistical Analysis

The following calculation formulas were used to evaluate diagnostic performance: sensitivity (%) = [true positive number/(true positive number + false negative)] × 100, specificity (%) = [true negative/(true negative + false positive)] × 100, and healing rate of esophageal fistula (%) = (number of healed cases/total number of cases) × 100. Survival time of patients with esophageal fistula was defined as the interval from fistula onset to death or last follow-up. Kaplan–Meier survival analysis was performed to construct patient survival curves and calculate the median survival period. When we confirmed the influence of age, thickness, and length of the esophageal fistula, the Kolmogorov–Smirnov test was performed to determine whether the data were normally distributed. If the data were normally distributed, the results were expressed as the means ± standard deviations. The two groups were compared using the Student *t*-test. IBM SPSS Statistics for Windows, version 22 (IBM Corp., Armonk, NY, USA), was used for statistical analysis, and *P* < 0.05 was considered statistically significant.

## Results

A total of 206 patients with pathologically confirmed inoperable esophageal cancer were enrolled. The basic characteristics of patients are summarized in Table 1.

**Table 1:** Basic characteristics of patients.

**Patient demographics**	
Number of patients	206
Age(years):mean (range)	66 (43–87)
**Gender**	
Male	167
Female	39
**Esophageal lesion site**	
Cervical	20
Thoracic	186
**Histopathology**	
Squamous carcinoma	205
Adenocarcinoma	1
Other	0
**Clinical stages**	
T^*^	
T1	9
T2	56
T3	76
T4	65
**N**	
N0	47
N1	102
N2	44
Nx	13
**M**	
M0	181
M1	9
Mx	16
**Concurrent chemotherapy**	
Yes (mean age, years)	127 (63)
No (mean age, years)	79 (72)
**Chemotherapy regimens**	
Fluoropyrimidine and Platinum	26
Taxus and Platinum	7
Tegafur (S1)	78
Cisplatin	8
Other	8

* *T stages: the length of lesions was determined by barium esophagogram*.

During radiotherapy, 10 cases of esophageal fistula were detected or suspected based on oral meglumine diatrizoate esophagogram findings. Eight cases were confirmed by CT and fibroesophagoscopy, including three cases of esophageal tracheal fistula, three cases of esophageal bronchial fistula, and two cases of esophageal mediastinal fistula, making the final incidence rate 3.88% (8/206 patients). All patients with confirmed esophageal fistulas received intervention by discontinuing radiotherapy, fasting, and receiving high parenteral nutrition and anti-inflammatory medication. After interventional treatment, the fistulas in five patients were healed after 1–2 weeks of treatment, and the patients resumed radiotherapy to completion. The healing rate of esophageal fistulas was as high as 62.5% (Table 2). Therefore, the rates of sensitivity and specificity of oral meglumine diatrizoate esophagogram in screening for esophageal fistulas during radiotherapy were 100 and 98.99%, respectively (Table 3). We additionally found that esophageal fistulas could occur at any time during radiotherapy, with ~75% of esophageal fistulas observed to occur during the 15th through 20th fractions (32.25–43 Gy) of radiotherapy. There were five patients with complete dysphagia, which was diagnosed by barium esophagography before radiotherapy, and they received high parenteral nutrition until they could consume a liquid diet. No patients required nasogastric tube feeding.

**Table 2:** Screening of esophageal fistula by oral meglumine diatrizoate esophagogram during radiotherapy.

**Screening of esophageal fistula during radiotherapy**	
**Esophageal fistula**	
Yes	8 (3.88%)
No	198 (96.12%)
**Time of esophageal fistula**	
10F^#^	2 (25%)
15F^#^	4 (50%)
20F^#^	2 (25%)
**Therapeutic effect of intervention on esophageal fistula**	
Fistulas recovered and radiotherapy completed	5 (62.5%)
Fistula not recovered and radiotherapy not completed	3 (37.5%)

# *F, fractions of radiotherapy*.

**Table 3:** Diagnostic performance evaluation is as follows.

**Diagnostic test**	**CT^!^/Fiberesophagoscopy**		**Total**	**Sensitivity**	**Specificity**
	**+**	**–**			
+	8	2	10	100%	98.99%
–	0	196	196		
Total	8	198	206		

! *CT: Spiral enhanced computer tomography*.

The average thickness of esophageal lesions in patients with esophageal fistulas was 0.29 cm thicker than that in patients without esophageal fistulas, but this difference was not statistically significant (*P* = 0.105).The occurrence of esophageal fistulas was significantly related to the length of the lesions; the length of the lesions in patients with and without esophageal fistulas was 8.77 and 5.98 cm, respectively (*P* = 0.001). We found that age was also significantly related to the occurrence of esophageal fistulas (*P* = 0.026) (Table 4).

**Table 4:** Factors influencing esophageal fistula.

	**Groups (*n*)**	**Mean**	**S.D**.	***F***	***P***
Age (years)	A (196)	67	9.682	0.280	0.026
	B (8)	59	10.029		
Lesion thickness^&^ (cm)	A (196)	1.52	0.534	0.762	0.105
	B (8)	1.81	0.355		
Lesion length^@^ (cm)	A (196)	5.98	2.286	0.372	0.000
	B (8)	8.77	2.760		

& *Lesion thickness: The maximum esophageal thickness of the lesion is shown on CT*.

@ *Lesion length, Length of lesion in barium esophagogram*.

**Figure 1:**
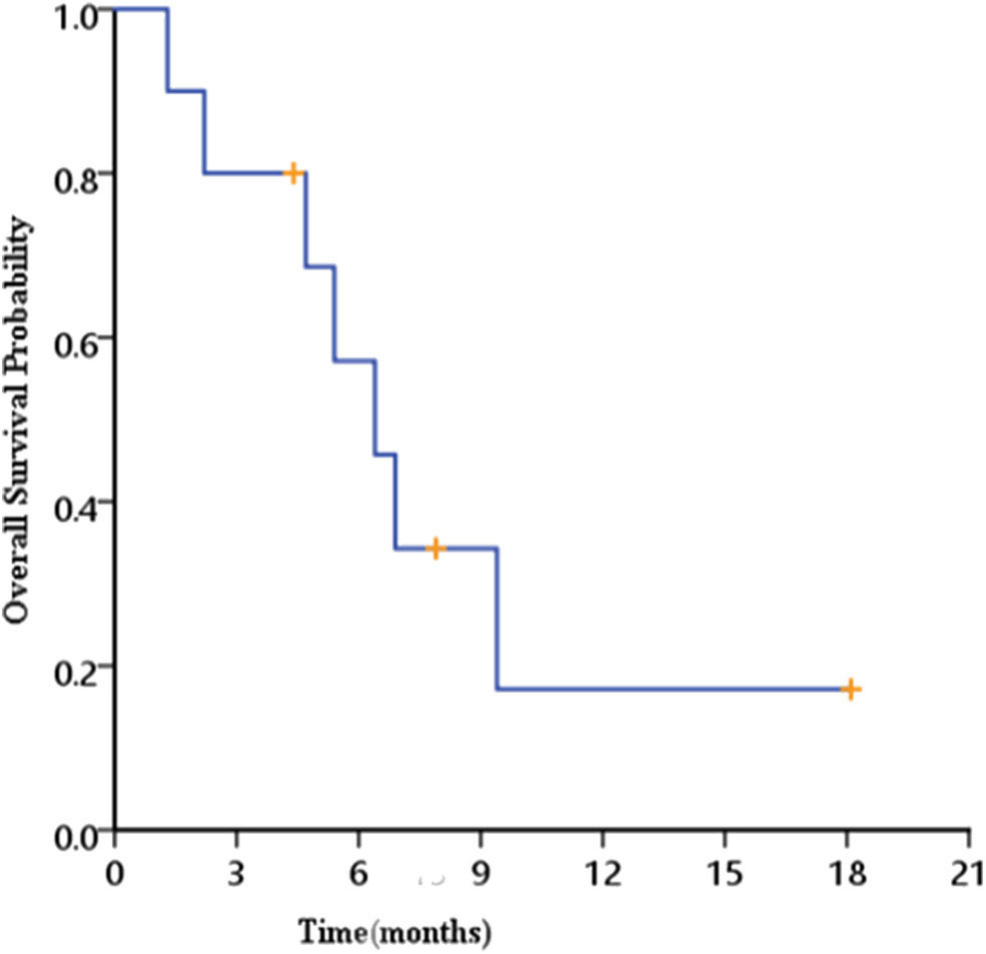
Overall survival of patients.

The median follow-up time was 18.1 months (6–30 months). Seven patients died; four patients had tumor-related diseases, and the other three patients died of non–tumor-related diseases. Kaplan–Meier survival analysis showed that the median survival period for esophageal fistula patients was 6.4 months (1.3–18.1 months) (Figure 1).

## Discussion

Esophageal fistula is a serious complication of radiotherapy treatment for esophageal cancer. It may result in fever, cough, choking due to cough, chest pains, upper gastrointestinal bleeding, and other symptoms. Some patients may even die of systemic infection and massive bleeding (14). Its prognosis and therapeutic response are very poor, with multiple studies showing that the median survival time was only 2–3 months (6–9). The latest research in 2020 reported median survival data of only 3.63 months (5), making early detection very important.

Previous studies have indicated that barium esophagogram was advantageous for treating small fistulas (10). However, the use of barium sulfate has been shown to have serious side effects, aggravating the symptoms of fistula patients. In fact, the drug's instruction label clearly states that it should not be used to treat patients with esophageal tracheal fistulas. Moreover, its rates of sensitivity and specificity in diagnosing esophageal fistulas were only 45.5 and 97.8% (11), respectively. We actually had more patients who completed treatment than was reported in the previous literature (13). We had 103 patients who completed the treatment, and four of these cases developed esophageal fistula. The incidence (3.88%) of esophageal fistula was lower than our previous estimate of 8%. We recounted the sample size and increased the sample size to 206 patients after obtaining the approval of the ethics committee. Compared with the poor diagnostic performance of barium esophagogram, the diagnostic performance of meglumine diatrizoate esophagogram is much greater, with its rates of sensitivity and specificity reaching 100 and 98.99% in our study, respectively. In this study, 10 cases of esophageal fistula were detected or suspected based on oral meglumine diatrizoate esophagogram findings, and eight were confirmed by CT and fibroesophagoscopy. The other two cases were deemed negative by CT and fibroesophagoscopy. One of those two patients died 1 month later of acute upper gastrointestinal bleeding after radiotherapy. The other patient ultimately presented with esophageal fistula after completing treatment and receiving placement of an esophageal stent. Patients who have a suspected sign of esophageal fistula via oral meglumine diatrizoate esophagogram but not later confirmed by CT and fibroesophagoscopy need to be followed up closely as they have a high risk of serious complications.

For patients with locally advanced esophageal cancer, with or without esophageal fistula, the continuation and completion of radiotherapy are very important (13, 15). Early detection of esophageal fistulas and timely intervention can increase completion rate of radiotherapy, which may improve the prognosis and prolong the survival period of patients with esophageal fistulas. Our study confirmed that even if esophageal fistulas occurred, 62.5% of patients can recover and continue to complete their radiotherapy schedule through timely and effective intervention. The median survival time can reach 6.4 months (1.3–18.1 months), which is significantly higher than findings from previous studies (5–9). The longer survival time can be attributed to the following two factors: early detection and timely treatment of esophageal fistula and the completion of radiotherapy or chemoradiotherapy treatment by more than half of the patients, which resulted in control of tumor growth.

In this study, we found that 11 (5.3%) patients had cases of mild diarrhea, most of which occurred 3 to 4 h after ingesting the oral contrast agent, and all of which were relieved within 24 h without drug treatment. Meglumine diatrizoate is a hypertonic iodine contrast agent that can transfer the liquid between tissues and blood vessels to the intestinal cavity. As a result, the intestinal contents are diluted, stimulating peristalsis of the small intestine and ultimately causing diarrhea symptoms. Some studies have even found that meglumine diatrizoate can reduce edema of the intestinal wall, promoting the relief of intestinal obstruction (16, 17). Although the taste of meglumine diatrizoate has been deemed poor compared to other iodine-enhanced imaging agents (18), no significant differences in side effects or diagnostic performance have been observed (19, 20). More importantly, the price of meglumine diatrizoate in China is lower than that of other iodine contrast agents, making it cost-effective and more suitable for the routine screening of patients with esophageal cancer.

This study determined that it is advantageous to screen for esophageal fistulas via oral meglumine diatrizoate esophagogram. However, the incidence of esophageal fistulas during radiotherapy in our study was 3.88%, whereas other radiotherapy centers have seen inconsistent rates between 3 and 20% (21–23). Oral meglumine diatrizoate esophagogram should be used in esophageal cancer patients with high risk of esophageal fistula during radiotherapy. Chen et al. (6) found that the occurrence of esophageal fistula after radiotherapy was related to T4, CRT. Moreover, Wang et al. (24) found that the occurrence of esophageal fistula was related to T4 and tumor size and thickness. However, our study did not find that there was a significant correlation between esophageal fistula presentation and esophageal wall thickness. Our study instead found that the occurrence of esophageal fistulas was related to the length of the esophageal lesions and age. Sex, synchronous chemotherapy, presence of lymph nodes, and distant metastasis were not found to be related to esophageal fistula occurrence. A possible explanation is that the proportion of esophageal fistulas in this study was relatively low, the sample size was small, and the statistical analysis failed to show significant differences.

Despite these inconsistencies, the latest research by Xu et al. demonstrated useful results in identifying the individual risk stratification of patients (25). They analyzed the characteristics of 136 patients who developed esophageal fistulas during or after radiotherapy and found that Eastern Cooperative Oncology Group Performance Status (ECOG PS), body mass index, T4, N2/3, and re-radiotherapy were independent factors for esophageal fistulas. The researchers created and externally validated the risk nomogram of esophageal fistulas associated with radiotherapy, which will ultimately aid individual risk stratification of patients with esophageal cancer who develop esophageal fistulas. Using the nomogram of this study, 80% of patients with high scores (>180 points) will develop esophageal fistulas. Our study results recommend that those patients with high scores undergo oral meglumine diatrizoate esophagogram once a week during radiotherapy.

## Conclusion

Oral meglumine diatrizoate esophagogram has high sensitivity and specificity in screening for esophageal fistulas during radiotherapy and is associated with minimal side effects. Early diagnosis and timely intervention can significantly improve the prognosis and prolong the survival period of patients. It can be used to screen esophageal cancer patients with a high risk of developing esophageal fistulas during radiotherapy.

## Data Availability Statement

The raw data supporting the conclusions of this article will be made available by the authors, without undue reservation.

## Ethics Statement

The studies involving human participants were reviewed and approved by The medical ethics committee of the MianYang Central Hospital. The patients/participants provided their written informed consent to participate in this study.

## Author Contributions

XD: guarantor of integrity of the entire study and manuscript editing. RW, LG, and BT: study concepts and design. RW and LG: literature research. RW, LG, ZZ, DL, BH, HH, YL, ML, MX, YZ, and GF: data collection. RW, LG, and XD: data analysis. RW and LG: manuscript preparation. All authors read and approved the final manuscript.

## Conflict of Interest

The authors declare that the research was conducted in the absence of any commercial or financial relationships that could be construed as a potential conflict of interest.
